# Left Atrial Appendage Occlusion: Current Stroke Prevention Strategies and a Shift Toward Data-Driven, Patient-Specific Approaches

**DOI:** 10.1016/j.jscai.2022.100405

**Published:** 2022-07-13

**Authors:** Keegan Mendez, Darragh G. Kennedy, Dee Dee Wang, Brian O’Neill, Ellen T. Roche

**Affiliations:** a Harvard/MIT Health Sciences and Technology Program, Massachusetts Institute of Technology, Cambridge, Massachusetts; b Institute for Medical Engineering and Science, Massachusetts Institute of Technology, Cambridge, Massachusetts; c Department of Biomedical Engineering, Columbia University, New York, New York; d Henry Ford Health, Detroit, Michigan; e Department of Mechanical Engineering, Massachusetts Institute of Technology, Cambridge, Massachusetts

**Keywords:** computed tomography, imaging, left atrial appendage, left atrial appendage occlusion, stroke

## Abstract

The left atrial appendage (LAA) is a complex structure with unknown physiologic function protruding from the main body of the left atrium. In patients with atrial fibrillation, the left atrium does not contract effectively. Insufficient atrial and LAA contractility predisposes the LAA morphology to hemostasis and thrombus formation, leading to an increased risk of cardioembolic events. Oral anticoagulation therapies are the mainstay of stroke prevention options for patients; however, not all patients are candidates for long-term oral anticoagulation. Percutaneous occlusion devices are an attractive alternative to long-term anticoagulation therapy, although they are not without limitations, such as peri-implant leakage and device-related thrombosis. Although efforts have been made to reduce these risks, significant interpatient heterogeneity inevitably yields some degree of device-anatomy mismatch that is difficult to resolve using current devices and can ultimately lead to insufficient occlusion and poor patient outcomes. In this state-of-the-art review, we evaluated the anatomy of the LAA as well as the current pathophysiologic understanding and stroke prevention strategies used in the management of the risk of stroke associated with atrial fibrillation. We highlighted recent advances in computed tomography imaging, preprocedural planning, computational modeling, and novel additive manufacturing techniques, which represent the tools needed for a paradigm shift toward patient-centric LAA occlusion. Together, we envisage that these techniques will facilitate a pipeline from the imaging of patient anatomy to patient-specific computational and bench-top models that enable customized, data-driven approaches for LAA occlusion that are engineered specifically to meet each patient’s unique needs.

## Highlights

• Description of LAA anatomy and pathophysiology pertinent to AF-related stroke.
• Prevention strategies to decrease stroke risk associated with atrial fibrillation.
• Advances in imaging, planning, modeling and manufacturing in LAA occlusion.
• Future perspective on a pipeline towards patient-specific LAA occlusion.

## Central Illustration

**Figure:**
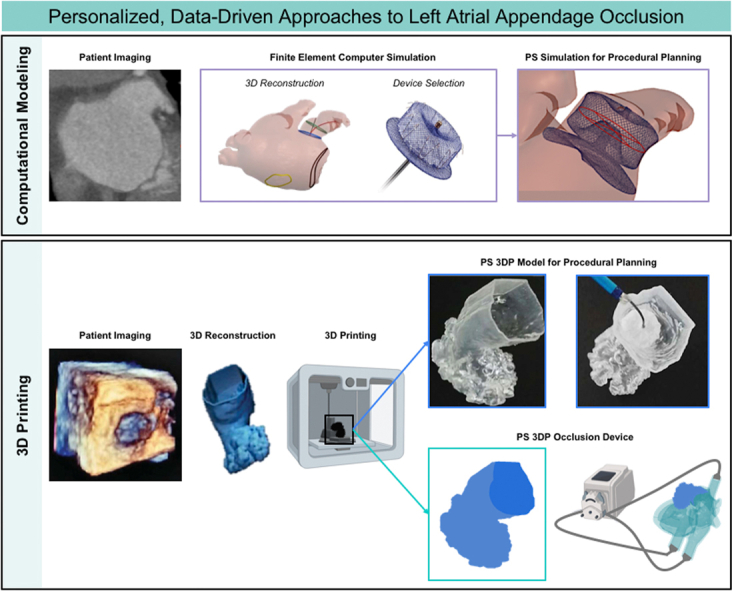

## Introduction

The left atrial appendage (LAA) is a complex structure with unknown physiologic function protruding from the main body of the left atrium. The LAA has been hypothesized to serve as a fluid reservoir^1^ and decompression chamber^2^ during various phases of the cardiac cycle. In patients with atrial fibrillation (AF), the left atrium does not contract effectively. Insufficient atrial and LAA contractility predisposes the LAA morphology to hemostasis and thrombus formation. Thus, in patients with AF, this underlying condition predisposes the patients to an increased risk of cardioembolic events. Oral anticoagulation (OAC) therapies are the mainstay of stroke prevention options for patients. However, not all patients are candidates for long-term OAC. In this state-of-the-art review, we evaluated the anatomy of the LAA as well as the current pathophysiologic understanding and stroke prevention strategies used in the management of the risk of stroke associated with AF.

## LAA: structure, function, and pathology

### LAA

**Figure. 1:**
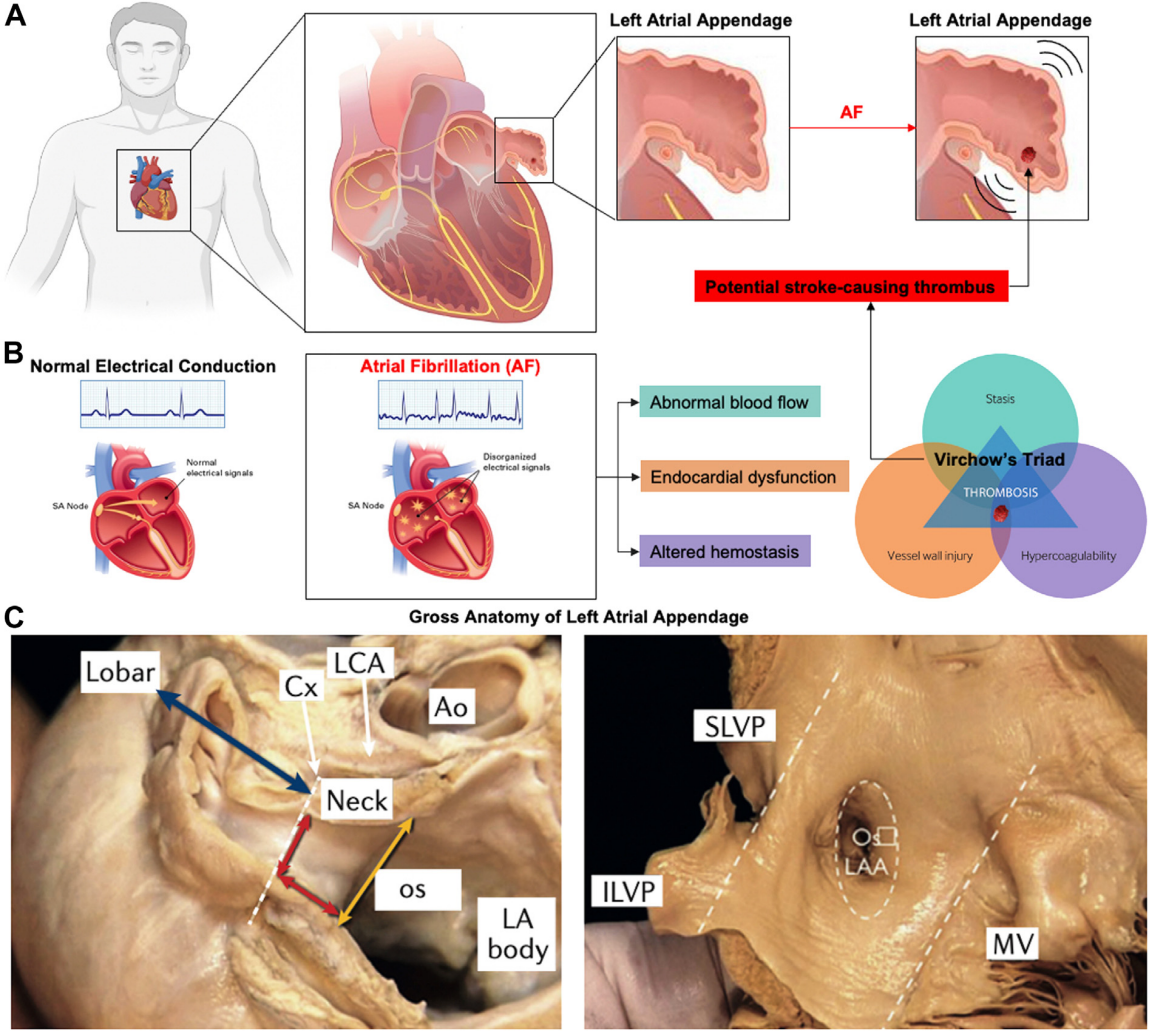
**Location, anatomy, and morphology of the left atrial appendage (LAA).** (**A**) Location of the LAA within the heart and the representation of clot formation during atrial fibrillation. **(B**) Atrial fibrillation (AF) is caused by abnormal electrical activity. The central feature of AF is rapid and uncoordinated atrial activity. Disordered electrical propagation causes disorganized stimulation of the myocardium and subsequent arrhythmic contractions. AF decreases contractility, resulting in blood stasis and diminished peak flow velocities. AF is also associated with endothelial damage, fibrosis, and inflammation, especially within the LAA, which leads to a prothrombotic and hypercoagulable state. This association is consistent with the Virchow triad, which synthesizes the pathogenesis of clot formation in the LAA in patients with AF: abnormal blood flow, endocardial dysfunction, and altered hemostasis. (**C**) The LAA is located near the atrioventricular groove between the left ventricle and the pulmonary artery trunk, with the base close to the proximal left circumflex artery. The LAA is divided into 3 anatomic regions: the opening or ostium, neck, and lobar region. The ostium can be teardrop-shaped, round, elliptical, foot-like, or triangular, and its diameter can range from 10 to 40 mm. The lobar region usually consists of 2 lobes and is the most spatially complex region of the LAA, with heavy trabeculations and pectinate muscles. The length and width of the neck, as well as the number of lobes, vary considerably. The LA body is smooth-walled. AF, atrial fibrillation; Ao, aorta; ILPV, inferior left pulmonary vein; Cx, circumflex artery; LA, left atrium; LCA, left coronary artery; MV, mitral valve; os, ostium; SLPV, superior left pulmonary vein. Reproduced from Nishimuraet al,^3^ 2019, and Caliskan et al,^4^ 2017.

The LAA is a complex morphologic structure (Figure 1).^2^ It is typically divided into 3 anatomic regions: the ostium (orifice), neck (landing zone for transcatheter devices), and lobar region.^4^ Externally, the LAA has the appearance of a flattened tubular structure with crenellations and usually has 1 or 2 bends, which terminate in a pointed tip.^5^ There is significant variation in the size, morphology, and internal anatomy of the LAA among patients^6^, ^7^, ^8^ as well as heterogeneity in its interaction with adjacent cardiac and extracardiac anatomy. The LAA volume has been reported to range from 0.7 to 9.2 mL, with the ostial dimensions ranging from 10 to 40 mm and the LAA lengths ranging from 16 to 51 mm.^9^ The ostium is typically elliptical, with the mean long-axis diameters in the range of 16-23 mm and the mean short-axis diameters in the range of 10-17 mm.^10^, ^11^, ^12^ Postmortem studies have demonstrated the LAA to be a multilobed structure in 80% of cases.^13^ Such variability in LAA anatomy precludes the ability of a one-size-fits-all concept for device development.^8^

The LAA has multiple anatomic attributes related to thrombus formation in the setting of atrial arrhythmias. First, the LAA is a contractile reservoir that functions as a decompression chamber during periods of high left atrial (LA) pressure (ventricular systole) because of its increased distensibility compared with the rest of the left atrium.^2^ As an actively contracting structure, the dysfunction of the LAA can lead to thrombus formation. In patients without AF, the average LAA contractile velocity ranges from 60 to 83 cm/s, with an average LAA filling velocity of 47-60 cm/s.^14^ Second, LAA contractility decreases with age, whereas the incidence of AF increases with age; thus, the risk of thrombus formation increases significantly over time.^15^ Third, in addition to its active role in cardiac hemodynamics, several studies have indicated that the LAA has neurohormonal properties, such as the regulation of thirst through stretch-sensitive receptors, modulation of intravascular volumes, and regulation of hemodynamics by the endocrine release of atrial natriuretic peptide.^16^, ^17^, ^18^ To date, the potential impact of atrial arrhythmias on the neurohormonal properties of the LAA and the associated risk of thrombus formation has not been well understood.^19^ Hence, the risk and incidence of LAA thrombus formation are a significantly more complex mechanism than previously understood.

### Thrombus formation in the LAA

Thrombus formation in the LAA largely occurs secondary to a combination of abnormal LAA contractility, hemostasis, and blood flow in the LAA. In general, 3 flow patterns have been described in the LAA: type I, characterized by a regular biphasic emptying pattern, occurring in sinus rhythm; type II, characterized by a saw-tooth emptying pattern, occurring in some patients with AF; and type III, characterized by no active emptying, occurring most often in patients with AF.^3^ During type III, the LAA acts as a static pouch and is consequently associated with the highest incidence of spontaneous echo contrast and thrombosis.^20^ In patients with AF, the LAA can oscillate at rates faster than 300 bpm, which leads to substantially reduced outflow. A reduced LAA peak outflow velocity is considered one of the strongest predictors of increased thromboembolic risk.^21^ Patients with chronic AF tend to have an increased maximal appendage size, a near-zero appendage ejection fraction, and decreased filling and emptying velocities, all of which contribute to blood stasis and increased thrombotic risk.^22^ In patients with mitral stenosis and AF, there also tends to be chronic pressure overload, which strongly decreases LAA contractile function.^23^ The combination of mitral stenosis and AF may additionally predispose patients to the risk of thrombus formation in the left atrium.^24^ Thrombus formation is a complex mechanism triggered by many factors, including prothrombotic processes, such as angiotensin-mediated processes and increased adhesivity of the endocardium.^25^ The pathophysiology of thrombus formation can be described as Virchow triad, which consists of the following: (1) endothelial dysfunction, (2) abnormal blood stasis, and (3) altered hemostasis. Although the precise etiology is unknown, AF is associated with endothelial damage, fibrosis, and inflammation, especially within the LAA, which leads to a prothrombotic and hypercoagulable state, consistent with the Virchow triad and the pathophysiology of thrombogenesis.^26^, ^27^, ^28^

### Risk factors for LAA-derived thrombus formation

The CHA_2_DS_2_-VASc scoring system is currently used to provide stroke risk stratification estimates for patients with AF.^29^^,^^30^ Two points are awarded for previous stroke history and age >75 years, and 1 point is assigned for each of the following if present: previous congestive heart failure, hypertension, diabetes, vascular disease, age of 65-74 years, and female sex.^31^ However, this scoring system does not consider the presence or absence of LAA contractility.

In patients with sinus rhythm, the LAA acts as a highly contractile muscular sac. Patients with sinus rhythm with a thrombus in the LAA showed a decrease in the LAA ejection fraction from 55% to 18% and a decrease in the peak velocity from 48 to 24 cm/s. In patients with AF, the LAA displays passive filling and emptying because of compression due to the adjacent left ventricle. In patients with AF or flutter with contrast and/or a thrombus in the LAA, the LAA behaves like a static pouch. In a study of 75 patients with various cardiovascular diseases who were referred for transesophageal echocardiography (TEE) examination, Li et al^32^ found that patients with poor LAA function had a higher incidence of spontaneous echo contrast or thrombus formation in the LAA. If the patients had lost LAA contractile function, almost no identifiable flow in the LAA was recorded, and a poor LAA ejection fraction and a low emptying peak velocity were found. The static LAA became a location for blood stasis where thrombus formation occurred. In the Stroke Prevention in Atrial Fibrillation III TEE study, which included patients with AF, 17% of patients with contraction velocities of ≤20 cm/s had thrombi present compared with 5% of patients with higher velocities.^33^ LAA vortices and blood flow regulation likely contribute to the different rates of thrombus formation in patients with varying LAA anatomy. Specifically, an increase in the number of LAA lobes correlates with low LAA emptying velocity and suggests that complex LAA morphology, owing to the increased number of lobes, is likely to induce blood stasis and potentiate thrombus formation.

## Current strategies to prevent LAA-related thrombi

**Figure. 2:**
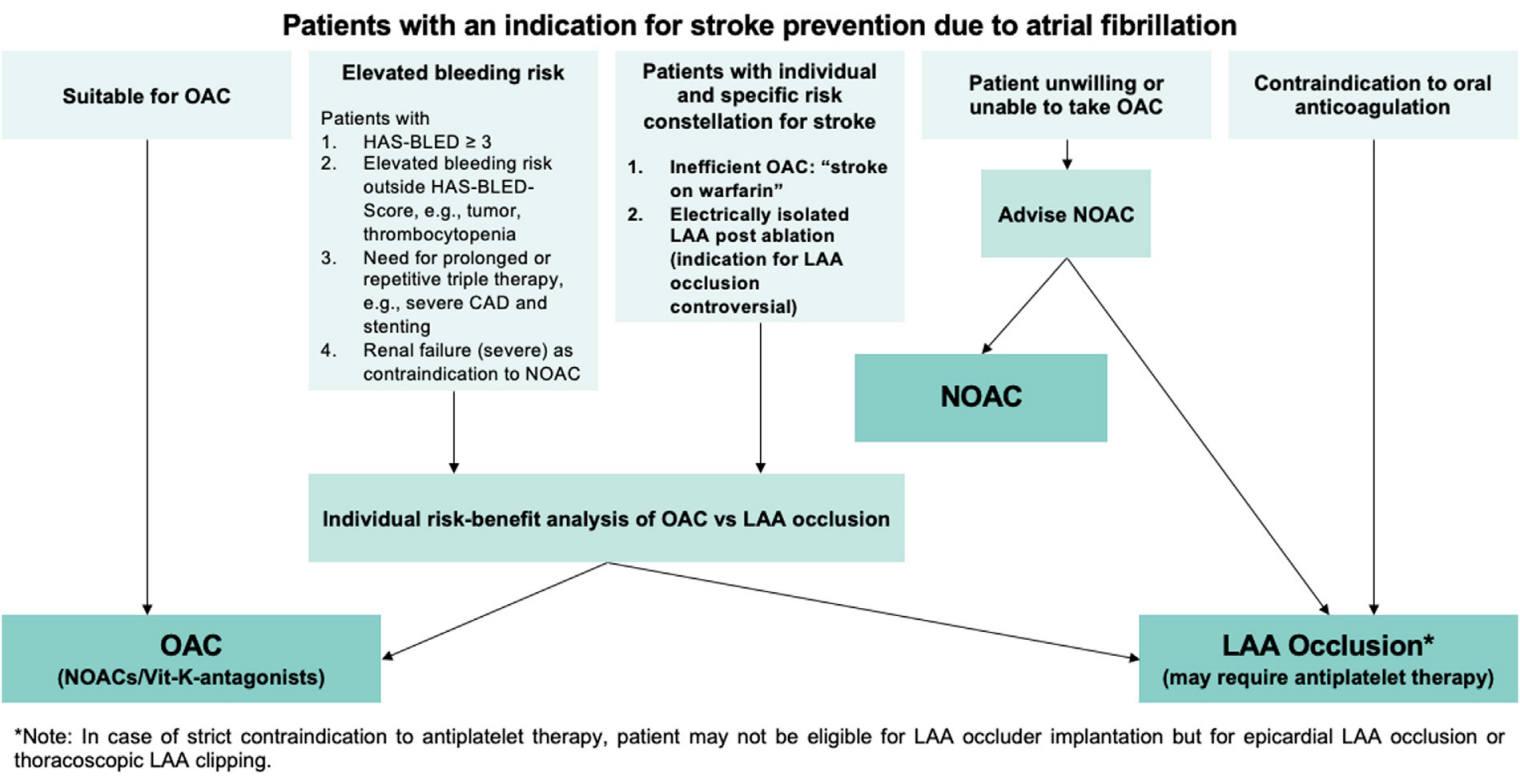
**Decision tree for left atrial appendage closure in patients with an indication for stroke prevention due to atrial fibrillation**. CAD, coronary artery disease; HAS-BLED, scoring system developed to assess 1-year risk of major bleeding in people taking anticoagulants for atrial fibrillation (Hypertension, Abnormal renal and liver function, Stroke, Bleeding, Labile INR, Elderly, Drugs or alcohol); LAA, left atrial appendage; NOAC, novel oral anticoagulant; OAC, oral anticoagulant; Vit-K, vitamin K. Reproduced from Glikson et al,^34^ 2020.

One of the first approaches toward the prevention of AF-related stroke involves the elimination of underlying arrhythmia, achieved by converting abnormal rhythm disorder into sinus rhythm. However, antiarrhythmic drugs, surgical interventions, and catheter ablations are only partially successful in restoring stable sinus rhythm in patients with AF. Therefore, additional stroke prevention strategies are often necessary for patients with AF (Figure 2). OAC therapy is the mainstay of stroke prevention therapy, with direct oral anticoagulants (DOACs) now preferred over warfarin because of a lower risk of bleeding.^35^ Nonetheless, there remains an inherent risk of bleeding due to DOACs, along with other challenges associated with long-term anticoagulation therapy, such as compliance and undertreatment, that result in a subset of patients being unable or unwilling to remain on long-term OAC therapy.^36^ Nonpharmacologic or mechanical strategies that locally prevent the development of thrombi within the LAA are an alternative to OAC in these patients. In addition, the LAA can be removed surgically by excision or exclusion. However, surgery is not always an option because increased frailty, high blood pressure, diabetes, and other comorbidities commonly associated with an increased risk of stroke in the population with AF are the causes for patients to be deemed as poor surgical candidates. The risks associated with invasive surgery have led to the development of percutaneous occlusion devices that can be deployed via the transcatheter approach to eliminate the need for long-term anticoagulation therapy.

### Pharmacologic anticoagulation therapy

Large, randomized trials have demonstrated that the use of dose-adjusted vitamin K antagonists (VKAs) for OAC significantly reduces the risk of stroke in patients with AF.^37^, ^38^, ^39^, ^40^, ^41^ Despite their clinical potency, a significant proportion of patients with AF do not receive OAC therapy with VKAs^42^^,^^43^ because of medication or lifestyle limitations. Studies involving controlled cohorts on warfarin therapy showed that patients remained in a therapeutic range only on 50%-70% of monitored days.^44^

The latest guidelines for the management of AF recommend OAC as the first-line treatment option for anticoagulation.^45^ Concerns about the use of VKAs have led to the development of non-vitamin-K–dependent oral anticoagulants (NOACs), which directly inhibit either factor Xa (apixaban, rivaroxaban, and edoxaban) or thrombin (dabigatran). NOACs have proven to be safer (lower rates of major hemorrhage) and more effective (lower rates of stroke, systemic embolism, and death due to cardiovascular causes) than OACs in large clinical trials and have now become the standard of care for many patients.^44^^,^^46^, ^47^, ^48^ However, NOACs still have several drawbacks, such as high costs, the risk of bleeding, drug-drug interactions, and the need for dose adjustment in elderly patients or those with low weight and reduced renal or liver function. With roughly 30%-50% of patients with AF ineligible or unwilling to receive anticoagulation therapy,^49^ there is a clear need for an alternative means for stroke prevention.

### Surgical excision or exclusion

Despite the favorable risk-to-benefit ratio of NOACs compared with that of warfarin, an inherent risk of bleeding accompanies all anticoagulation drugs. Therefore, there is a need for alternative therapies for the prevention of AF-related stroke. The LAA can be excluded from the systemic circulation by occluding its orifice or excising the entire body of the appendage. The LAA can be mechanically excluded using multiple surgical techniques: epicardial suture ligation, endocardial suture occlusion, and excision and epicardial suturing. Excision is considered the most effective and definitive surgical technique, with epicardial and endocardial suture occlusions associated with high rates of persistent, residual, or recurrent connections between the LAA and left atrium.^4^

The AtriClip LAA Exclusion System (AtriCure) allows for epicardial occlusion with concomitant open cardiac surgery or as a minimally invasive, stand-alone thoracoscopic procedure.^50^ AtriClip consists of a sterile, implantable, self-closing clip that is applied to the epicardium and positioned at the base of the appendage. The clip itself it made of 2 parallel titanium tubes with elastic nitinol springs that are wrapped in a knit-braided polyester sheath. The device acts by applying uniform pressure around the base of the appendage, which prevents flow between the appendage and the atrium and subsequently results in the atrophy of the appendage and organization by dense fibrous tissue.^51^ The base of the LAA is first measured, with 4 clip sizes available (35, 40, 45, and 50 mm). Once the clip is in an optimal position, it is closed and released from the deployment tool manually. Once deployed, it is difficult to reposition the clip, requiring careful manual opening of the clip and reinsertion on the deployment tool. The EXCLUDE trial (AtriCure Exclusion of the LAA in Patients Undergoing Concomitant Cardiac Surgery) revealed successful intraprocedural LAA exclusion in 65 out of 70 patients (92.9%), and 60 out of 61 patients (98.4%) had successful exclusion, which was confirmed using computed tomography (CT) angiography or TEE.^52^ However, the AtriClip exclusion device is only available in 4 sizes (35, 40, 45, and 50 mm), thus precluding a patient cohort with an LAA width of <29 mm or >50 mm from being eligible to undergo this procedure.

#### Challenges associated with surgical excision or exclusion

Despite evidence that the surgical management of the LAA is effective, there are significant risks associated with the incomplete closure of the LAA. A landmark study published in 2008 evaluating different surgical techniques in patients who underwent surgical left atrial appendage occlusion (LAAO) from 1993 to 2004 found a higher success rate of LAAO after excision (73%) than after suture exclusion (23%) and stapler exclusion (0%).^53^ However, a meta-analysis of attempted LAAO by surgical methods found incomplete closure in 34%-45% of cases,^54^ which has been shown to increase the risk of stroke from 2% to 24%.^53^^,^^55^^,^^56^ Hence, because of the presence of a higher rate of incomplete surgical closure, transcatheter options have been developed to decrease the incidence of peridevice leakage.

### Percutaneous LAAO devices

Because there are significant risks associated with incomplete LAAO due to surgical excision or exclusion methods, research and development efforts have shifted toward focusing on percutaneous occlusion devices (LAAO devices) that can be deployed in a minimally invasive fashion to substantially reduce or eliminate the need for long-term anticoagulation therapy.

The ideal characteristics that should be achieved by all LAAO devices include the following:

1. Safe and efficacious with proven ability to reduce strokes
2. Capable of minimally invasive deployment
3. Ability to treat a wide spectrum of LAA shapes and sizes
4. Long-term stability without the development of residual leakage or device embolization (DE)
5. No interference with any surrounding intracardiac structures
6. Avoidance of device-related thrombi
7. Safely minimizes the duration of postimplantation anticoagulation therapy

**Figure. 3:**
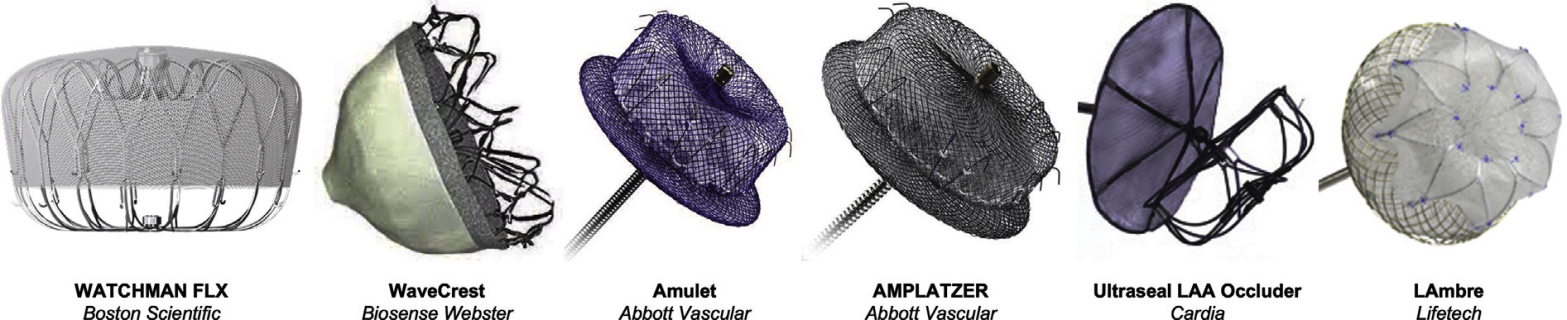
**Commercially available, CE-mark approved percutaneous left atrial appendage occlusion devices.** Adapted from Glikson et al,^37^ 2020. CE, Conformité Européenne.

The catalog of marketed percutaneous LAAO devices has been extensively reviewed elsewhere,^57^, ^58^, ^59^, ^60^ with some of the most common devices shown in Figure 3. Watchman FLX (Boston Scientific) and Amulet (Abbott) are the 2 current LAAO devices commercially approved by the Food and Drug Administration for use in the United States in patients with nonvalvular AF. The Watchman device is a self-expanding nitinol cage with fixation bards and a permeable polyester fabric covering the surface of the device at the atrial side. LAAO with the Watchman device is noninferior to warfarin therapy in the prevention of ischemic stroke and systemic thromboembolism and is associated with lower rates of hemorrhagic stroke, bleeding, and death.^61^ A network meta-analysis of the comparative effectiveness of interventions for stroke prevention in patients with AF ranked the Watchman device first with respect to the reduction of all-cause mortality and the risk of stroke compared with warfarin and the leading NOACs.^62^ Additionally, a cost-efficacy analysis showed that LAAO is cost effective compared with treatment with warfarin or dabigatran (NOAC).^63^ Since these trials, however, DOACs have been shown to be superior to warfarin and have a class I indication for use in patients with nonvalvular AF. A network meta-analysis that included 19 randomized controlled trials (RCTs), with a total of 87,831 patients with AF receiving anticoagulants (warfarin or DOACs), antiplatelet therapy, a placebo, or LAAO (Watchman device), showed that LAAO was superior to antiplatelet therapy or the placebo and comparable with DOACs in the prevention of mortality and stroke or systemic embolism.^64^ The recent PRAGUE-17 trial (Left Atrial Appendage Closure vs Novel Anticoagulation Agents in Atrial Fibrillation), the first-ever RCT to compare DOACs with LAAO (Watchman or Amulet) in high-risk patients with AF, found that LAAO was noninferior in the prevention of major AF-related cardiovascular, neurologic, and bleeding events in patients at a high risk of stroke and an increased risk of bleeding.^65^

Although LAAO devices are viable alternatives for patients with AF who are not candidates for long-term anticoagulation, the success of these devices may be attenuated by issues such as peri-implant leakage and device-related thrombosis (DRT). In addition, the anatomic variability of the LAA has not been completely resolved by the myriad of devices currently available, with certain LAA morphologies not suitably matched in structure to a percutaneous occlusion device.

#### Peri-implant leakage

Currently available LAAO devices have standard circular geometries (Figure 3), whereas the ostium of the LAA is typically elliptical and displays high interpatient variability. It is this mismatch in geometry that can lead to incomplete LAAO and an increased risk of residual peridevice leakage. Peridevice leakage or flow has been defined as residual flow of any size, detected using TEE. Minor peridevice leakage is common after LAAO with the Watchman device, with a leakage of <5 mm considered minor. A peridevice leakage of >5 mm is considered significant and can be managed with OAC and serial imaging follow-up.^66^ A recent review found that the incidence of severe peridevice leakage following LAAO is nonnegligible and highlighted the paucity of clear, evidence-based management strategies for the management of these leaks.^66^ Leaks have been reported in as many as 32% and 59% of patients receiving the Watchman and Amplatzer devices, respectively^8^^,^^67^; however, an interim report from the SURPASS study with the new-generation Watchman device, Watchman FLX, showed lower rates of peridevice leakage.^68^ The results of SURPASS, which included 16,048 patients who received Watchman FLX, showed that after implantation, 95.3% of the patients had no peridevice leakage and 99.1% had no leakage or had a leakage of <3 mm. At 45 days after implantation, these percentages were 82% and 95.4%, respectively. Although it has been debated whether the presence of leakage following LAAO device implantation is associated with thromboembolic risk, studies have suggested that leakage following surgical ligation is associated with increased thromboembolic events.^21^^,^^53^ Early LAAO procedures used 2-dimensional (2D) imaging, which led to device undersizing, which was associated with increased incidences of peridevice leakage and DE. With the introduction of CT preprocedural planning and the identification of optimal device oversizing compression ranges of 10%-20% (allowing for device-specific anatomic variations), there has been a significant decrease in these procedurally related adverse events.^69^ Without this oversizing measure, the device has the potential to embolize, which is associated with high morbidity and sometimes requires the retrieval of the device.^70^

#### Device-related thrombosis

**Figure. 4:**
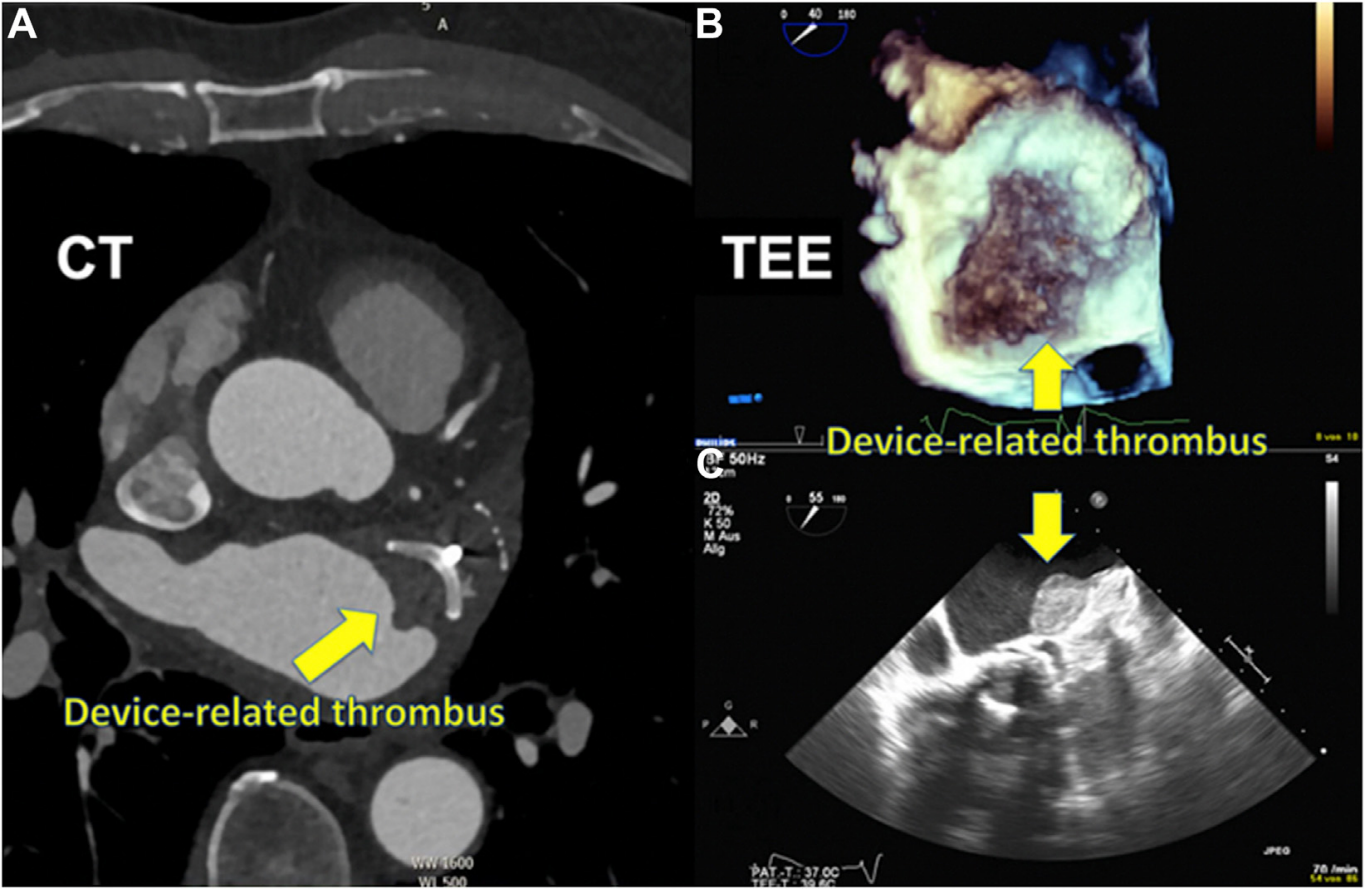
**Device-related thrombosis (DRT) detected during imaging follow-up**. (**A**) DRT detected using cardiac computer tomography in a 75-year-old patient 590 days after left atrial appendage closure. (**B, C**) DRT in 3- and 2-dimensional transesophageal echocardiography in an 87-year-old patient 226 days after left atrial appendage closure. CT, computed tomography; TEE, transesophageal echocardiography. Reproduced from Sedaghat et al,^71^ 2021.

Although it is known that there is a risk of DRT (Figure 4) following the implantation of LAAO devices, data on the incidence and prediction of DRT remain limited. Current reports have suggested that DRT occurs in 3%-4% of patients following LAAO and that DRT is associated with a significantly increased risk of ischemic events.^72^, ^73^, ^74^ A retrospective study performed in 2018 involved an analysis of 469 patients with AF who underwent LAAO (272 received Watchman devices and 197 received Amplatzer devices).^74^ The study found that the incidence of DRT in patients who underwent LAA imaging was 7.2% per year, with older age and a history of stroke being predictive factors for thrombus formation on the devices. Dual antiplatelet therapy and OAC therapy were found to be protective factors. The study shed light on the potential negative impact of DRT, with thrombi on the device being an independent risk factor for ischemic stroke and transient ischemic attacks during follow-up. In a more recent effort to gather data on the clinical outcomes of patients experiencing DRT relating to LAAO, Sedaghat et al^71^ analyzed data from 156 patients diagnosed with DRT after LAAO. After 2 years of follow-up, the patients with DRT were found to have a high risk of ischemic stroke (13.8%) and mortality (20.0%). The identification of specific risk factors for DRT has remained elusive because of variable patient, anatomic, technical, and pharmacologic factors, as described in multiple small studies.^72^, ^73^, ^74^^,^^75^ Simard et al^76^ recently developed a DRT registry via a multicenter collaboration aimed at accessing the outcomes and predictors of DRT in an effort to improve risk stratification and optimize strategies to mitigate DRT after LAAO. A total of 711 patients (237 with DRT and 474 without DRT) were included in the study. They found that DRT occurs in ∼5% of cases following LAAO and is associated with a higher risk of ischemic events and major adverse cardiac events. Hypercoagulability disorders, renal insufficiency, nonparoxysmal AF, and a device implantation depth of >10 mm from the pulmonary vein limbus were found to be risk factors and more important predictors of DRT than the antithrombotic drug regimen employed for prophylaxis on discharge after LAAO. Following conversion to risk factor points, patients with 2 risk points for DRT had a 2.1-fold increased risk of DRT compared with those without any risk factors. Overall, this study confirmed that DRT after LAAO is associated with ischemic events and provided an important insight into patient- and procedure-specific factors associated with an increased risk of DRT that can aid in the risk stratification of patients referred for LAAO. An interim analysis from the SURPASS study using newer-generation Watchman FLX demonstrated that at 45 days after implantation, the rate of adverse events was 0.91% for death, 0.38% for stroke, 0.28% for ischemic stroke, 0.01% for systemic embolism, 0.23% for DRT, 0.03% for DE, and 3.55% for major bleeding.^68^ Longer-term clinical data comparing the DRT rates of early-generation LAAO devices with those of newer-generation devices are not yet available.

#### DE

DE, like DRT, is a complication of LAAO that can have substantial implications on patient morbidity and mortality. At present, there are limited patient-level data regarding the clinical outcomes of patients who experience DE. Murtaza et al^77^ recently performed a retrospective analysis of DE cases from worldwide multicenter experience to better understand the patient characteristics, timing, and clinical sequelae of DE. They included 103 cases in the study (59 received an Amplatzer cardiac plug, 31 received Watchman, 11 received Amulet, 1 received LAmbre, and 1 received Watchman FLX) and found that the incidence of DE was 2% (103/5000). They determined that DE occurred more commonly in the postoperative period than in the intraoperative period (61% vs 39%, respectively; *P* = .06) and that the most common location for embolization was the descending aorta (30%) and the left atrium (24%), followed by the left ventricle (20%). In most cases (75%), the device was retrieved percutaneously; however, surgical retrieval was often necessary for devices embolized to the left ventricle, mitral apparatus, and descending aorta. Major adverse events (including death) were significantly more in postoperative DE than in intraoperative DE (44.4% vs 22.5%, respectively; *P* = .03). Death occurred in 8 cases (8%). Operator-reported device and LAA size mismatch was the most identified factor associated with DE. Overall, the findings of this study suggested that DE is common, with a reported incidence of 2%. DE occurs more frequently in the postoperative setting, which is associated with a higher rate of complications, need for surgical retrieval, and mortality compared with intraoperative DE. These findings reiterate the importance of appropriate device sizing and highlight the need for postprocedural follow-up imaging to identify and minimize the complications of acute DE.

## Toward personalized occlusion of the LAA

Personalized medicine is an advanced health care delivery approach in which treatment is customized according to each patient’s unique needs.^78^, ^79^, ^80^ It has been shown that this targeted approach leads to better patient outcomes, with personalized therapies typically displaying more effective responses and higher safety margins than blunt, one-size-fits-all solutions. Structural heart interventions, such as LAAO, require a comprehensive understanding of cardiac pathophysiology. The LAA is a highly anatomically variable structure that comes in multiple shapes and sizes and displays a variety of convexities, concavities, lobes, and trabeculations. Such diverse patient anatomy is highly amenable to personalized medicine, in which each procedure is precisely tailored for each patient. Significant strides in personalized medicine, specifically in the field of structural heart disease, have been made possible using techniques such as 3-dimensional printing (3DP), computational modeling, and artificial intelligence.^81^ 3DP can decrease the early-operator learning curve for new technology adaptation,^82^, ^83^, ^84^, ^85^, ^86^, ^87^, ^88^, ^89^, ^90^, ^91^ computational fluid modeling can emulate dynamic physical and physiologic properties of cardiac pathophysiology,^92^, ^93^, ^94^, ^95^, ^96^ and the application of artificial intelligence has potential for patient-specific anatomic replica procedural simulation training.^97^, ^98^, ^99^, ^100^ In this section, we describe technologies reported in the literature that are being leveraged for more optimal and personalized LAAO procedural planning, device sizing, and device placement, as well as recent fabrication reports of novel occlusive devices that are patient specific (Central Illustration).

### Patient-specific procedural planning, device sizing, and device placement for LAAO

#### Risk stratification and prediction of thrombus formation

Computation modeling is performed using numerical analysis methods such as finite element analysis and computation fluid dynamics (CFD). CFD has proven to be a useful complementary technique for predicting the risk of thrombi based on LAA morphology and indicating optimal device sizing and placement for LAAO. Bosi et al^101^ used CFD to analyze the CT images of healthy LAAs. Their results indicated that velocity and shear strain rate decrease along the central axis of the LAA, from the ostium to the tip. They also found that after 4 cardiac cycles, the lowest washout of the contrast agent was observed in patients with the cauliflower morphology, and the highest washout of the contrast agent was observed in patients with the windsock morphology. These results agree with reports in the literature that suggest that the cauliflower morphology is associated with a higher risk of thrombosis.^102^ This study revealed the potential of CFD for supporting patient risk stratification for thrombus formation.

CFD can also be used to better understand blood flow patterns after LAAO and predict the risk of DRT for a given patient and device configuration. Mill et al^103^ recently used patient-specific flow simulations to identify key blood flow characteristics associated with DRT. The authors performed patient-specific flow simulations for 6 patients (3 with DRT and 3 without DRT) who underwent LAAO at a single center with the Amulet device. Following analysis of the simulations, the authors identified the most relevant in silico indices associated with DRT as the presence of stagnant blood flow, recirculation with low flow velocities (<20 cm/s) adjacent to the device surface, and regions of high flow complexity combined with low wall shear stress. These results highlight the potential for the use of CFD in patient-specific procedural planning (risk assessment and device positioning) to minimize the risk of DRT.

A major limitation of CFD is high sensitivity to numerical assumptions, such as boundary conditions and material properties, that are adopted to reduce computational costs and simplify the modeling process. The results of CFD models are only as good as the assigned boundary conditions and the resolution of the input geometries. The CFD model development process may also be limited by the lack of relevant reference solutions or experimental data for model verification and validation.^104^

#### Imaging-based computational modeling and 3DP

Numerous cardiac imaging techniques are currently used to assess the anatomy and size of the LAA, including 2D TEE, 3D TEE, and cardiac CT. At the core of these technologies is the ability to evaluate the LAA using 3D assessment of its anatomy and surrounding structures. TEE has remained the gold standard for intraoperative preprocedural assessment,^105^ given its 3D, high-definition capabilities of the right and left atria, atrial septum, and LAA anatomy. In the absence of available TEE interventional imaging physician resources to help guide LAAO procedures, intracardiac echocardiography (ICE)-guided LAAO is a secondary option for LAAO. The current real-world limitations to the large-scale adaptation of ICE-guided LAAO include prohibitive costs of 4-dimensional ICE catheters, supply-chain mismatch with hospital access to ICE catheters, and limitations of ICE imaging inferior to the sector fields of 3D TEE. Recent reports have also indicated a potential role of CT-integrated fluoroscopy. Studies have shown that the integration of cardiac CT and fluoroscopy is feasible and safe and may be useful in reducing radiation exposure, procedure duration, and the volume of contrast media compared with CT alone.^106^^,^^107^ In the preprocedural setting, CT has been demonstrated to provide additive value for LAAO procedural planning versus stand-alone LAAO with 2D TEE.^108^

Similarly, with the rise of multiplanar and 3D reconstruction of the LAA using CT technology, multiple CT imaging-based software tools have been developed to aid in implanting physician teams’ understanding of the LAA anatomy. Virtual and physical 3D CT imaging-based computational models have been used to simulate device deployment into patient-specific cardiac anatomies, predict the optimal placement strategy, and determine optimal device sizing to reduce the potential for peri-implant leakage. Multiple simulation technologies exist, including OsiriX (Pixmeo), Mimics Enlight (Materialise), 3mensio (Pie Medical Imaging), and FEops HEARTguide (FEops NV). We describe a subset of these technologies that have been used in clinical practice in more detail below.

**Figure. 5:**
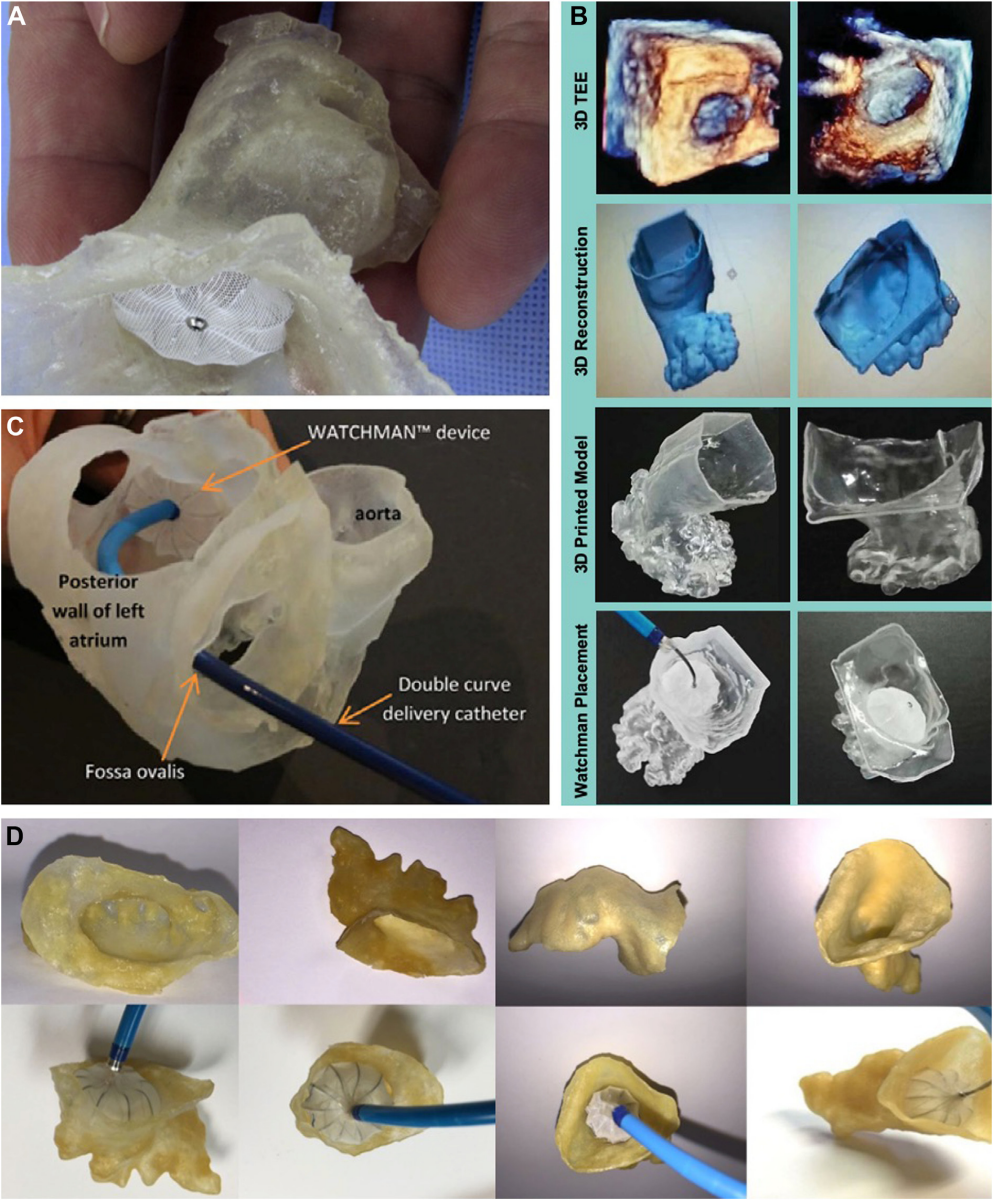
**The use of 3-dimensional (3D) printed models in left atrial appendage occlusion.** (**A**) The Watchman device placed within a flexible 3D-printed model demonstrates the clinical utility of 3D printing for device sizing and avoiding procedural complications. (**B**) 3D-printed models of the left atrial appendage using real-time 3D transesophageal echocardiographic data for assistance with physician planning and decision making. (**C**) 3D-printed model of a patient’s specific left atrial and left atrial appendage anatomy for assistance in the bench-test selection of catheter curvature for device implantation. (**D**) Computed tomography-based 3D-printed models for preprocedural planning in left atrial appendage device closure. Adapted from Otton et al,^109^ 2015; Liu et al,^110^ 2016; Wang et al,^69^ 2016; and Obasare et al,^111^ 2017.

In 2015, Otton et al^109^ reported left atrial appendage closure guided by personalized 3D-printed cardiac reconstruction (Figure 5A). Prior to the procedure, multidetector CT of the left atrium and atrial appendage was performed. The imaging data were segmented (Mimics; Materialise) and 3D printed in a rubber-like material to simulate atrial mechanical properties (Tango Plus Material, Stratasys Objet Connex 500 printer, and Stratasys). Watchman devices in multiple sizes (21, 24, and 27 mm) were deployed in the model, which was reimaged using clinical CT. The imaged 3D-printed LAA models with the Watchman devices were analyzed to determine the anatomic deformation for each device. Using the patient-specific, 3D-printed model and procedural simulation, the appropriate device size (24 mm) was selected and deployed, without any incidents.

Liu et al^110^ used 3D-printed models of the LAA based on real-time 3D TEE for preoperative reference prior to occlusion with the Watchman device (Figure 5B). They 3D printed models of the LAA from 8 patients with AF who underwent LAAO with the Watchman device and used the models for preoperative reference and procedural simulation. All 8 device sizes predicted by the 3D-printed models were consistent with those placed during the actual operation compared with only 7 of 8 devices predicted by preprocedural 2D TEE. Additionally, it was reported that the 3D-printed models could predict operating difficulty and the presence of peri-implant leakage.

Wang et al^69^ sought to examine the impact of 3D CT-guided procedural planning on the early-operator learning curve for the Watchman device (Figure 5C). LAAO with the Watchman device was performed in 53 patients. Preprocedural case plans were generated based on CT studies with the recommended device size, catheter selection, and C-arm angle for optimal deployment. For each patient, the CT data were segmented (Mimics) and 3D printed. A Watchman device was implanted ex vivo into the 3D-printed LAA to test fit the device and approximate the device landing zone. The authors found that the device selection was 100% accurate using CT imaging. Based on 2D TEE measurements alone, 62.3% of the cases (33/53) would have required larger devices. Based on 3D TEE measurements, 52.3% of the cases (28/53) would have required larger devices. The 3D TEE measurements would have inappropriately excluded 10 patients from Watchman implantation entirely. This study demonstrated that CT-based case planning allows a comprehensive and customized patient-specific LAA assessment that is accurate and may facilitate the reduction of the early Watchman implantation learning curve.

In agreement with the findings of Wang et al,^69^ comparative studies have suggested that device sizing is more accurate when 3D-printed LAA models based on preprocedural CT are used than when those based on TEE are used (Figure 5D).^112^, ^113^, ^111^, ^114^ Procedures that used a 3D-printed model based on CT imaging were associated with improved device selection and procedural efficiency (reduced procedure time, anesthesia time, and fluoroscopy time). Devices sized using a model based on CT imaging were also associated with a lower probability of peridevice leakage.^111^ Thus, the use of CT-informed, 3D-printed models of patient-specific LAA anatomy may prove clinically useful for more accurate device sizing and placement. Similarly, a 2021 study that reviewed 485 Watchman implantations at a single center compared the outcomes of using additional CT preprocedural planning (328 cases, 67.6%) with those of using stand-alone TEE guidance (157 cases, 32.45%) for LAAO and found that additional preprocedural planning using CT was associated with a higher successful device implantation rate, shorter total procedural time, and less frequent change of device sizes.^108^ Additionally, in the PRO3DLAAO RCT (Prospective, randomized comparison of 3-dimensional computed tomography guidance versus TEE data for left atrial appendage occlusion), the Henry Ford team found that CT-guided LAAO case planning was associated with improved device selection accuracy and procedural efficiency.^114^

**Figure. 6:**
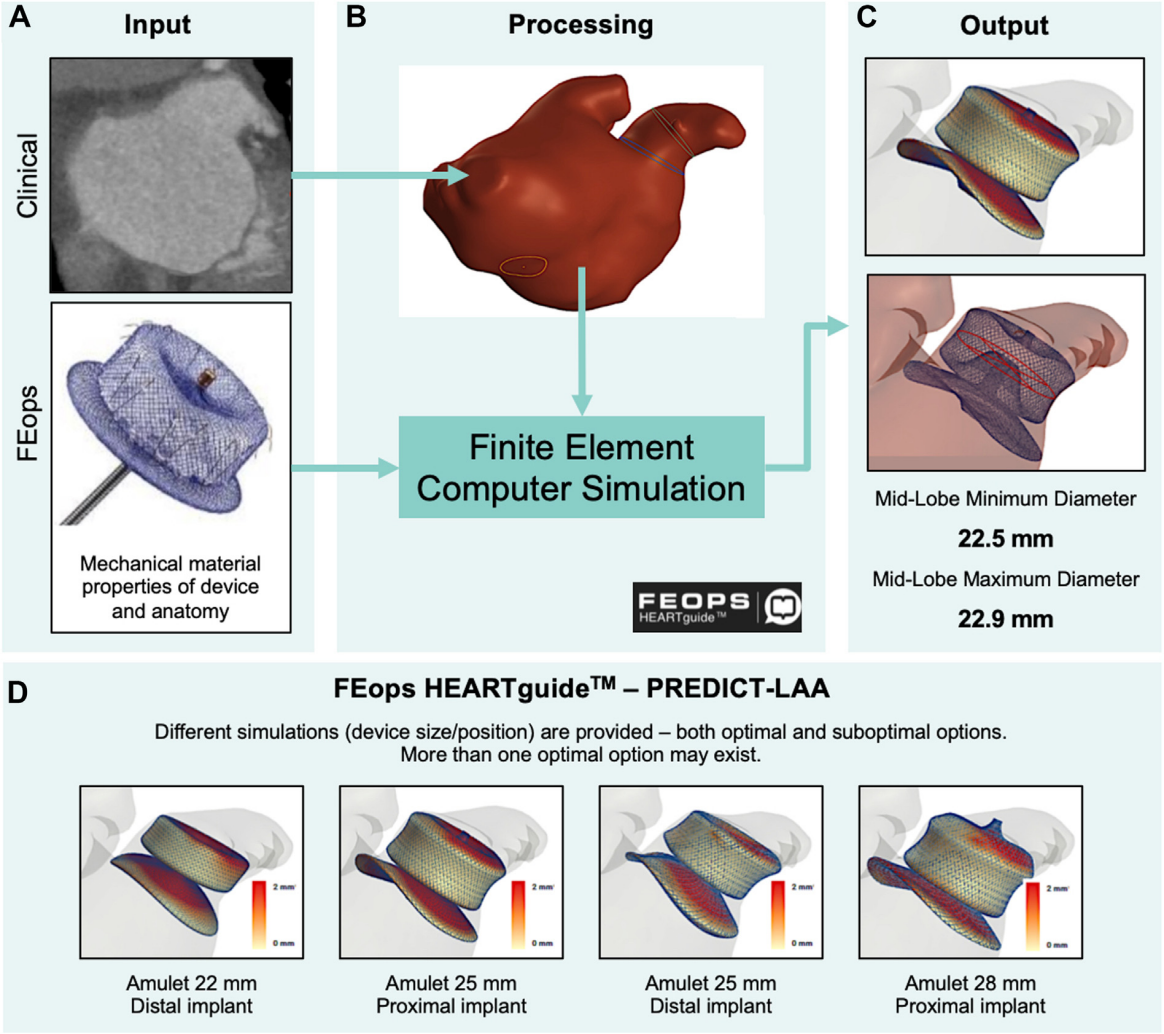
**FEops HEARTguide workflow and simulations.** Example of computed tomography-based virtual 3-dimensional planning software for patient-specific procedural planning. (**A**) Preprocedural computed tomography scan is uploaded on a web-based platform. (**B**) Image processing to extract 3-dimensional patient-specific anatomical reconstruction and landmarks for the procedure. Patient-specific reconstruction, in combination with the device model, serves as input for computational finite element analysis. (**C**) Model outputs several options in terms of device size and position, including left atrial appendage wall apposition plots, deformation visualization, and measurements. (**D**) Use of simulation output in clinical practice: for a single patient, different simulations in terms of device size and position are provided to the operator to help guide decision making before the procedure. Scale bar: white color indicates perfect apposition, and red color indicates gaps of ≥2 mm between the device and the walls. PREDICT-LAA, Value of FEops HEARTguide Patient-Specific Computational Simulation in the Planning of Percutaneous Left Atrial Appendage Closure With the Amplatzer Amulet Device. Reproduced from Garot et al,^115^ 2020.

Not all operators have access to CT-based 3DP technology. Hence, CT-based virtual 3D planning software products have been actively developed in the LAAO market. Materialise (Leuven), among other 3D modeling providers, has developed LAAO software packages for physicians to use for LAAO preprocedural planning. Other software companies have additionally developed simulation features evaluating potential deformation changes associated with different LAAO device landing zones. The FEops HEARTguide was developed to simulate the deployment of common LAAO devices into patient-specific anatomy for preprocedural planning (Figure 6). LAAO devices were implanted virtually into patients’ CT scans of the heart. Virtually implanted devices were directly compared with actual implants for device frame deformation and LAA wall apposition, with real-life patient data extracted from postprocedural cardiac CT imaging. This CT software is now being tested in the prospective randomized PREDICT-LAA trial (Value of FEops HEARTguide Patient-Specific Computational Simulation in the Planning of Percutaneous Left Atrial Appendage Closure With the Amplatzer Amulet Device) to assess whether CT is of additive valve to LAAO.^116^^,^^115^ Boston Scientific has developed a similar CT-based planning software, WATCHMAN TruPlan CT Imaging Software, that enables the visualization and measurement of the structure of the heart to facilitate patient screening and preprocedural planning for LAAO using the Watchman device.^117^ As more and more 3D planning CT-based software is developed, physician implanting teams will have access to many toolkits to help facilitate LAAO procedural planning.

Although imaging-based simulations and 3DP have been shown to lead to better interventional planning by improving device sizing, elucidating appropriate landing zones in a patient-specific manner, and improving overall procedural efficiency, they can only be used with currently available occlusion devices. There still exists a lack of percutaneous occlusion devices that can adapt to and are suitable for all patients, no matter what their underlying LAA anatomy.

### Dynamic bench-top models for preprocedural planning and novel device testing and validation

Although we see that patient-specific, 3D-printed models of the LAA improve precision in the sizing and placement of occlusion devices, these models are static and fail to recapitulate the complex motion, fluid flow, and tissue mechanics of the beating heart. Furthermore, 3D-printed models fail to emulate the dynamic physical and physiologic principles governing cardiac function. Biomechanical tissue characteristics, fluid flow dynamics, anatomic deformation, and tissue integrity involve complex fluid-structure interactions. The successful development of LAAO technologies requires the appreciation of complex anatomic pathophysiology integrated with the mechanical deformation properties of occlusion devices for a highly sophisticated tissue-fluid-structure interaction.

Bench-top circulatory flow loops are designed to simulate the cardiovascular hemodynamics of physiologic systems. To accurately model the human cardiovascular environment without relying on prohibitively expensive and time*-*consuming in vivo, large-animal studies, 3 main experimental approaches have been described: the use of synthetic ventricles,^118^, ^119^, ^120^ the integration of passive excised biological samples into artificial in vitro setups,^121^, ^122^, ^123^, ^124^, ^125^ and the use of ex vivo beating heart models.^126^, ^127^, ^128^ Synthetic models allow for easily controlled and repeatable experimental conditions but fail to recapitulate the complex intracardiac physiology. High-fidelity intracardiac anatomy is important for evaluating the function and simulating the placement of intracardiac devices, such as LAAO devices.

Ex vivo beating heart models provide highly realistic cardiac physiology. However, their price, complexity in model setup, and limited longevity (<1 day) because of muscle stiffening and decay prevent their widespread adaptation.^129^ In vitro approaches that use passive biological samples ensure the preservation of cardiac anatomy; however, to generate flow, the ventricular chamber is connected through the apex of the heart to an external pumping system.^123^^,^^124^ This pumping mechanism creates an altered, nonphysiologic fluid dynamic field inside the left ventricle. Currently, there is no valid bench-top model (synthetic or biological) that accurately mimics the complex motion, fluid flow, and anatomic features of the heart.

Scientists have tried multiple bench-top models for cardiac stimulation. Park et al^129^ developed a bio-hybrid approach that combines both organic and synthetic components. They demonstrated the fabrication of a biomimetic ventricular model that is composed of organic endocardial tissue and an active, soft robotic myocardium that drives the motion of the heart. Their model represents the anatomic details of intracardiac structures while also recreating physiologic cardiac motion using soft robotics. This innovation represents an important advance toward the need for high-fidelity cardiac simulators for preclinical intracardiac device testing; however, this model focuses primarily on ventricular motion and is not designed for modularity in which multiple, patient-specific anatomies can be interchanged and represented.

**Figure. 7:**
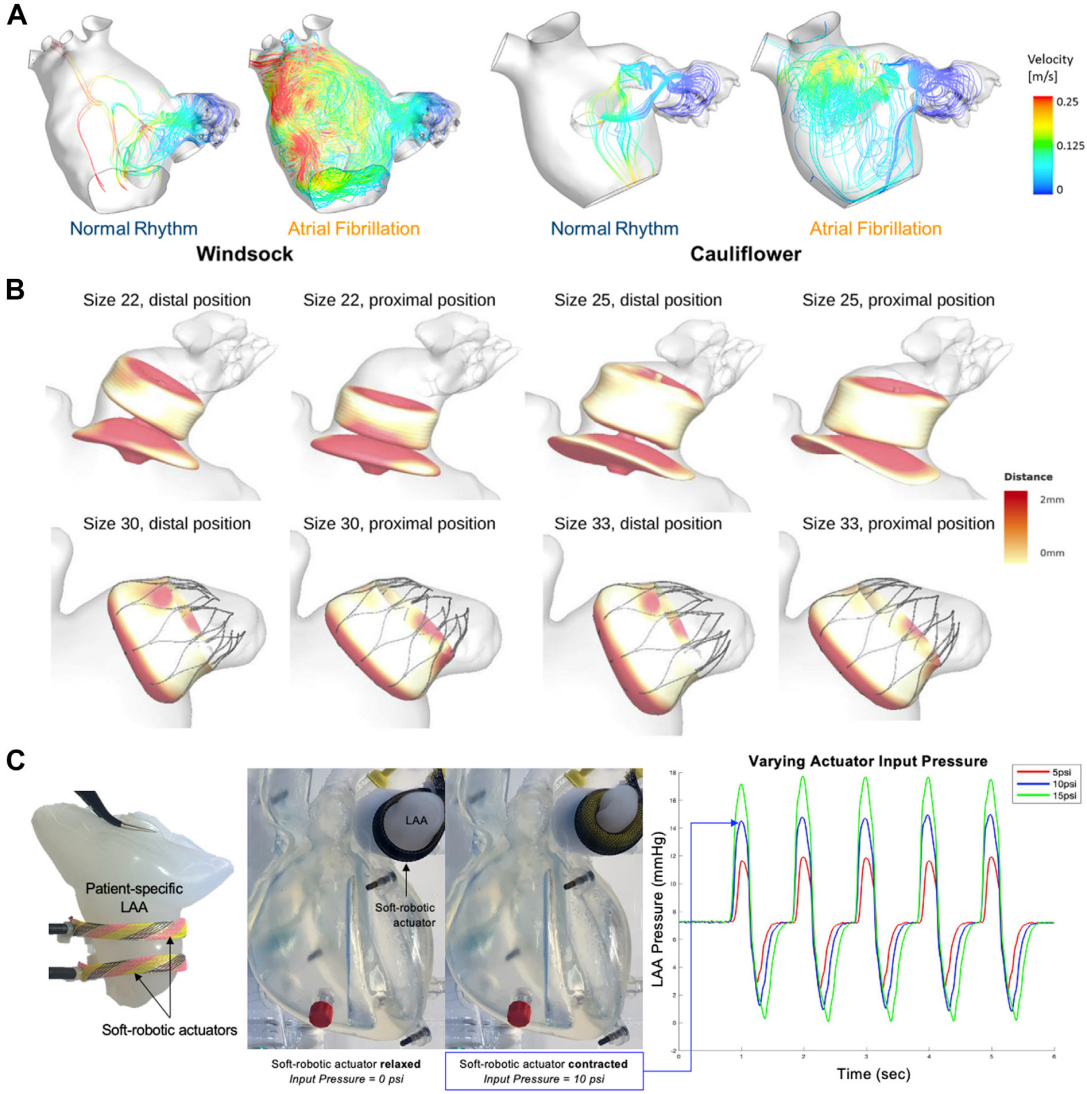
**Computational and bench-top models for left atrial appendage occlusion procedural planning and device testing and validation.** (**A**) Computational fluid dynamic analysis of the left atrial appendage (LAA) to predict the risk of thrombosis. Instantaneous velocity streamlines passing through the LAA orifice for both normal and pathologic atrial fibrillation condition. (**B**) Computational modeling to optimize preprocedural planning in left atrial appendage occlusion. Apposition plots for patients implanted with Amulet (top) and Watchman (bottom) devices. Different sizes and positions are virtually simulated, with white color indicating perfect apposition and red color indicating gaps of ≥2 mm between the device and the walls. (**C**) Design and development of bench-top circulatory model with interchangeable, patient-derived LAA geometries. Patient-derived silicone casting of LAA geometry that is incorporated into a cardiac simulator. Soft robotic actuators are used as artificial muscles to make the LAA contract cyclically. LAA pressure waveforms can be modulated by varying the actuation pressure and regime of the soft robotic actuators. LAA, left atrial appendage occlusion. Adapted from Bosi et al,^101^ 2018; Bavo et al,^130^ 2020; and Mendez et al,^131^ 2021.

More recently, the same group reported the design and development of a bench-top circulatory model with interchangeable, patient-derived LAA geometries that use soft robotics to drive the motion of patient-specific, silicone LAA models (Figure 7C).^131^ Mendez et al^131^ used patient imaging to 3D print patient-specific molds and cast silicone LAA models that can be incorporated into a bench-top circulatory loop and made active with soft robotic actuators. They generated physiologic and pathologic pressure waveforms by varying the actuation pressure of the soft robotic actuators. In the future, this dynamic, patient-specific model could be used as a tool for more realistic and physiologically relevant training and preprocedural planning and as a tool for the in vitro validation of computation modeling as well as novel device development and testing. Although this model replicates the LA anatomy and physiology, the lack of active contractility, relying instead on a peristaltic pump to generate pulsatile flow, could affect model performance because ventricular filling and emptying are known to influence LA and LAA hemodynamics. More generally, bench-top models are also limited by dependence on resistance valves and compliance chambers to mimic vessel material properties.

There is a clear role of computer simulation and modeling, 3DP, and advanced engineering techniques in the field of interventional cardiology (Figure 7).^101^^,^^130^^,^^131^ The future of LAAO will entail highly analytical, patient-specific procedural planning and device selection that requires the integration of clinical know-how with technical expertise in engineering and computer science.

### Fabrication of personalized LAAO devices

#### Toward improved device sizing

In recent years, there has been a surge in percutaneous LAAO device research and development that includes second-generation devices that aim to improve upon early devices by expanding device sizes and modifying device materials and shapes to lower the incidence of DRT. For example, the newer-generation Watchman device, Watchman FLX, and Amulet were developed with reduced metal exposure to decrease the incidence of DRT.^132^^,^^133^ Many new LAAO devices are currently in the research and development pipeline, with modifications to device anchoring and sealing approaches, which may offer improvements in the rates of DRT and peridevice leakage. Even with improved device sizing, it remains difficult to achieve a one-size-fits-all solution for percutaneous occlusion, given the significant heterogeneity in LAA geometry. Despite advancements in computational modeling techniques that can improve preprocedural device selection and target the implantation site, currently available devices will always yield a patient-device geometric mismatch that predisposes these patients to procedural risks and subtherapeutic outcomes. This fundamental mismatch cannot be overcome without novel device designs or occlusion techniques. To this end, we believe that future research efforts should focus on the development of a new class of customized LAAO devices that are based on and adapted to patient anatomy.

#### Personalized soft occlusion device from imaging-guided, 3D-printed mold

Rather than using imaging-derived, 3D-printed models of patient LAA geometry to inform device selection from currently available, off-the-shelf occlusion devices, 1 group recently used imaging-guided, 3D-printed models as molds to fabricate the first customized, patient-specific LAAO device.^134^ Robinson et al^134^ designed, developed, and tested in vivo a patient-specific design for a soft LAAO device by fabricating an inflatable silicone or polyurethane balloon using a 3D-printed mold derived from volume-rendered CT imaging of the LAA. The authors used an inflatable balloon made from a compliant material, with its geometry matching that of the anatomic morphology of the patient’s own LAA to minimize the strain on the balloon when expanded within the LAA and minimize the strain on the LAA tissue and neighboring structures. The fabrication of the CT image-guided, patient-specific soft LAAO device consisted of 4 key steps: first, image segmentation software was used to generate 3D renderings of LAA blood volume based on patient CT imaging; second, the LAA blood volume renderings were modified and processed using a computer-aided design to design the personalized occlusion device; third, molds for the device were designed and 3D printed; and fourth, the elastomeric device was molded from a customized blend of silicone and dip coated in polycarbonate urethane, a biocompatible surface coating that has been shown to be compatible with human endothelial cells. The compliance of the materials chosen helped the device to conform to the LAA anatomy upon inflation, which maximized the anchoring surface. The device was also integrated monolithically with a soft valve, which allowed the surgeon to fill or inflate the device without leakage once deployed within the LAA. The authors tested the inflation performance of the devices and found that the burst volumes were at least 127% higher than the necessary volume for complete LAAO. The authors concluded that the personalized device design enabled complete LAAO and prevented residual flow at much lower strains than noncustomized designs, demonstrating the advantages of patient specificity. Additionally, the patient-specific design resulted in smoother interfaces at the ostium, with smaller crevices, compared with the noncustomized design, suggesting that the patient-specific design was less likely to serve as a nidus for thrombus formation. This study highlights 2 major benefits of patient-specific occlusion devices: (1) reduced strain on the LAA and surrounding cardiac anatomy and (2) reduced peridevice leakage, which decreases the likelihood of DRT. In its current form, however, the wall thickness of this device is too large, preventing the device from fitting into a catheter for percutaneous delivery. Percutaneous delivery is preferred over thoracotomy with a pericardial incision (the surgical technique used to implant the device) to reduce procedural risk. Future work could also include a hemodynamic flow analysis following device deployment and inflation to understand the effects of LAAO with materials of varying mechanical properties and to different degrees of filling or strain.

#### Personalized occlusion device from imaging-guided stereolithography

Despite significant advancements in 3D additive manufacturing, most applications in the medical device space involve the use of hard or rigid materials. However, soft, compliant materials, such as elastomers, are more suitable for applications that involve contact with soft tissues. Such materials are especially desired for LAAO to better match native tissue properties, fully conform to complex LAA morphology, and minimize stress and damage to the LAA and neighboring anatomic structures. Building on previous work described above, Robinson et al^135^ more recently fabricated personalized soft LAAO devices using stereolithography (SLA) of elastomeric polyurethane resin. Again, the authors used patient CT imaging to inform the design of the occlusion device. Rather than fabricating devices using replica molding of silicones, the authors used high-resolution SLA printing to directly print the devices. Although SLA printing often involves resins that can be cytotoxic, especially uncured monomer species and photoinitiators used for curing, postprocessing steps can be taken to minimize the presence of these compounds prior to use in medical applications. Using this novel fabrication method, the authors were able to demonstrate a 40% reduction in device wall thickness, a 2-fold increase in device burst pressure, and improved anatomic matching to the native appendage structure. Further, such a fabrication method is highly cost effective, with a material cost of only $0.25/mL.^135^ Although this method showed improvements in matching the 3D-printed device to the patient’s anatomy, the long-term stability, hemocompatibility, and re-endothelialization of the device were not explored. Such considerations are crucial before bringing personalized devices into the clinical arena. Further work is also necessary to make the device more amenable for minimally invasive delivery because it currently requires an 18-F (6-mm diameter) catheter for deployment. Additionally, the device requires appropriate orientation during deployment to achieve the benefits of its patient-specific design, which may require additional operator training.

Taken together, these novel approaches for fabricating soft, personalized LAAO devices demonstrate the advantages of using additive manufacturing to produce highly patient-specific medical implants that resolve anatomy-device mismatch, overcoming the issues of conformability, peridevice leakage, and stress concentration that can perforate tissues. Depending on the material used, 3DP is generally considered cost effective for medical applications.^136^ More specifically, when deployed for procedural planning, training, and educational purposes, 3DP is highly cost efficient, enabling the rapid iteration and production of parts from a range of suitable materials that cost roughly $20-$100 per kilogram of material.^137^ Further work in device deliverability, long-term stability, and biocompatibility is warranted before successful translation into clinical practice.

## Conclusions

**Figure. 8:**
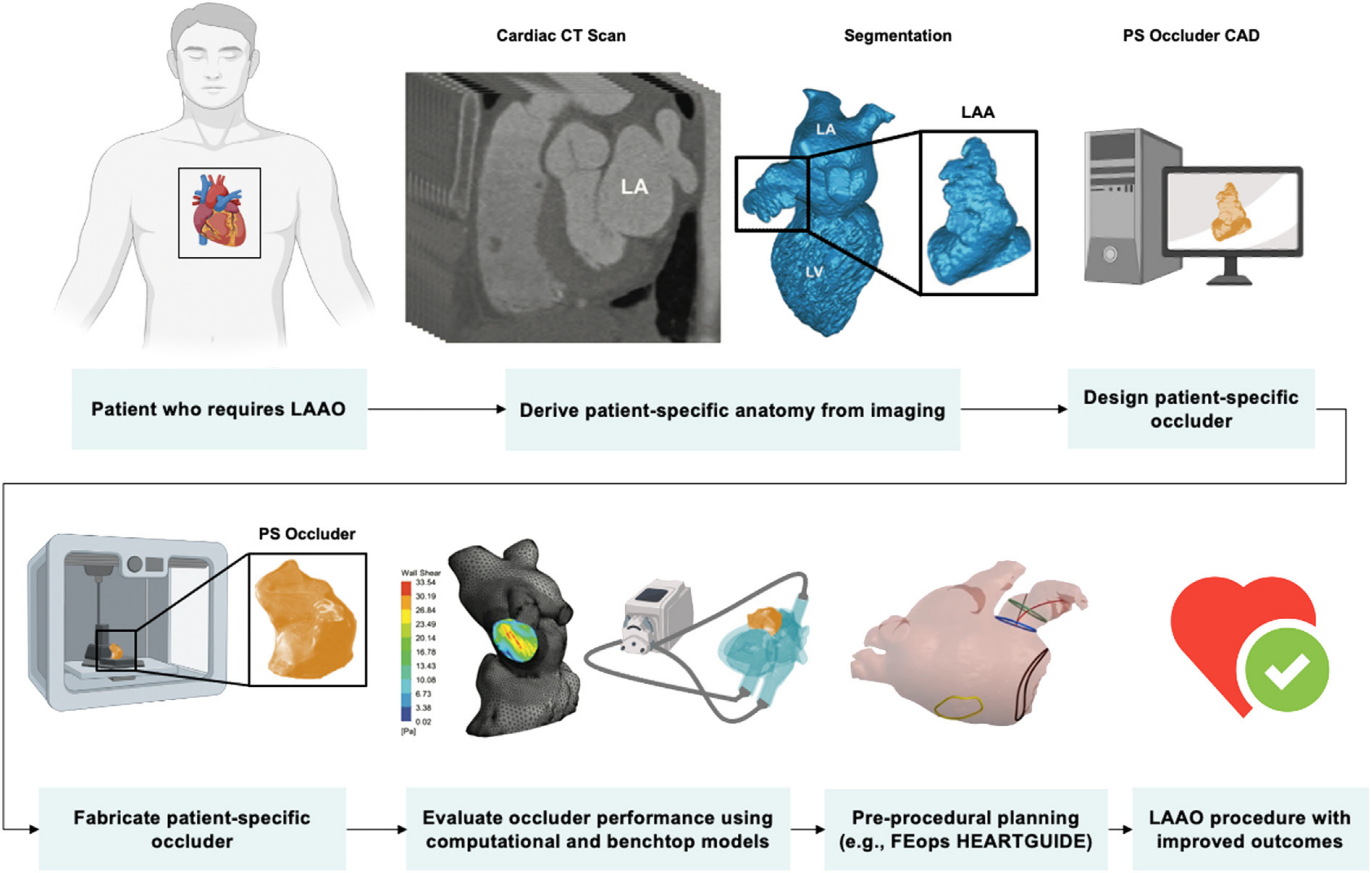
**Vision flowchart of personalized engineering approach for left atrial appendage occlusion.** Design, fabrication, validation, and implantation of a patient-specific left atrial appendage occlusion device. Adapted from Robinson et al,^135^ 2018; Gu et al,^138^ 2018; Senadeera et al,^139^ 2020; and FEops HEARTguide.^140^ CAD, computer-aided design; CT, computed tomography; LAAO, left atrial appendage occlusion; PS, patient-specific.

LAA morphology has high complexity and interpatient variability. Despite advancements in the field of LAAO device design and increased device opportunities for clinical use, there still exists a significant proportion of patients with anatomy-device mismatch. Anatomy-device mismatch can result in incomplete LAAO, peridevice leakage, and DRT, all associated with an increased thromboembolic risk. Such risks warrant a paradigm shift in LAAO device design. Recent advancements in computational modeling have led to improved preprocedural planning with currently available devices, helping implanting and interventional imaging physician teams choose both the optimal device size and target implantation location. With the development of novel fabrication techniques that use 3DP and SLA, research groups are now making strides toward a new class of medical implant: patient-specific devices custom fabricated based on patient data. The future of LAAO may improve patient outcomes owing to increased adaptation of data-driven approaches that use CT procedural planning and advanced additive manufacturing techniques for more patient-centric solutions (Figure 8).^138^, ^139^, ^140^

**Central Illustration:**
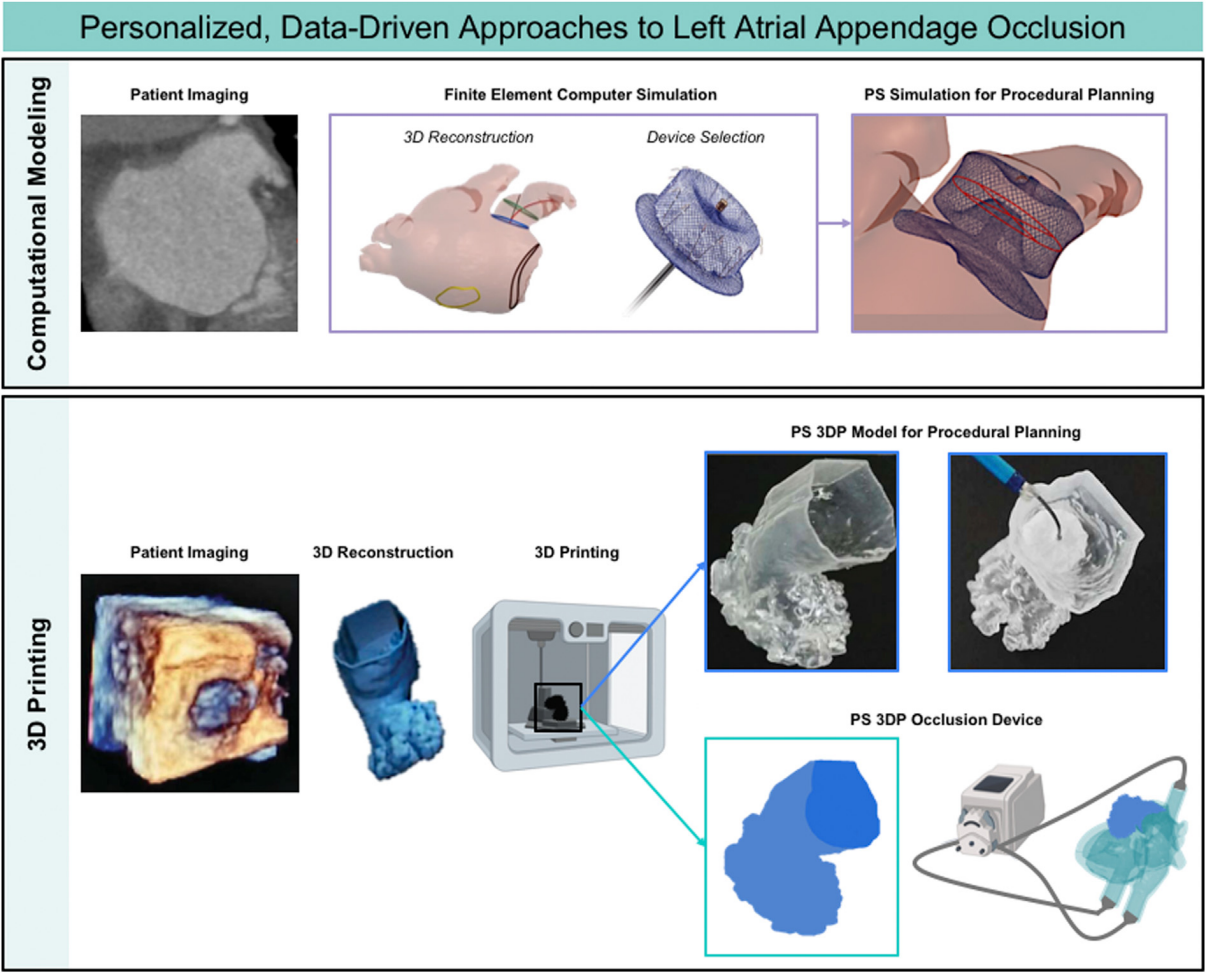
Personalized, data-driven approaches for left atrial appendage occlusion. Computational modeling technologies enable simulation of LAA occlusion device placement before procedures (**top**). Patient imaging can be coupled with 3D printing to create patient-specific LAA occlusion devices (**bottom**).

## Declaration of competing interest

Dr Wang is a consultant or advisory to Edwards Lifesciences Corporation, Boston Scientific Corp, Abbott, and Neochord Inc and receives funding grants from Boston Scientific Corp. Dr O’Neill is a consultant or advisory to Edwards Lifesciences Corporation and Abbott. Prof. Roche is a consultant to Holistick Medical and Surmodics and is on the board of directors for Affluent Medical and the scientific advisory board for Helios Cardiovascular. Ms Mendez and Mr Kennedy reported no financial interests.
